# Parasitic contamination of vegetables marketed in Arba Minch town, southern Ethiopia

**DOI:** 10.1186/s12879-019-4020-5

**Published:** 2019-05-14

**Authors:** Getaneh Alemu, Mohammedaman Mama, Direslgne Misker, Desta Haftu

**Affiliations:** 1 Department of Medical laboratory Science, Bahir Dar University, Bahir Dar, Ethiopia; 2 Department of Medical laboratory Science, Madda Walabu University Goba Referral Hospital, Robe, Ethiopia; 3 Department of public health, Arba Minch University, Arba Minch, Ethiopia

**Keywords:** Vegetables, Contamination, Parasite, Market

## Abstract

**Background:**

Consumption of unwashed, raw or unhygienically prepared fruits and vegetables act as potential source for the spread of various parasitic diseases. Moreover, the level of contamination and species of contaminant parasites vary from place to place because of variations in environmental and human factors. Therefore local determination of the level of contamination and associated factors is important for efficient intervention of infections acquired via those food items.

**Methods:**

A Cross-sectional study was conducted among purchased vegetables in selected markets of Arba Minch town from January to March, 2018. A structured questionnaire was used to capture data about factors associated with parasitic contamination of vegetables in the marketing phase. Selected vegetables were purchased and processed for examination of parasitic contamination using direct wet mount, iodine wet mount and modified zeihl Neelson staining following standard protocols. All data were analyzed using SPSS version 20.0.

**Results:**

Among 347 vegetable samples examined, 87(25.1%) were contaminated with at least one parasite species. Tomato (35.0%) was the most commonly contaminated vegetable while green pepper (10.6%) was the least contaminated one. *Entameoba histolytica/dispar* (29, 8.4%) was the commonest parasitic contaminant detected followed by *Giardia lamblia* (24, 6.9%) and oocyst of *Cryptosporidium* species (5.8%). Vegetable type (X^2^ = 13.5; *p* = 0.009) and source of vegetables (X^2^ = 24.1; *p* < 0.001) were significantly associated with parasitic contamination of vegetables.

**Conclusion:**

Parasitic contamination rate among marketed vegetables in the present study is significantly considerable. *Entameoba histolytica /dispar* was the most frequently detected parasite. We recommend to the local public health sector to establish a system for continuous monitoring of contamination of vegetables sold at local markets.

## Background

Consumption of fruits and vegetables is highly beneficial since they form the major component of a balanced diet [1]. Fruits and vegetables are important sources of carbohydrate, vitamins, minerals and fiber [2–4]. Considering such nutritional value, it is recommended that individuals should take a minimum of 400 g of fruits and vegetables per day. Fruits and vegetables also protect people from different non-infectious chronic diseases like cardiovascular problems [4, 5]. However; consumption of unwashed, raw or unhygienically prepared fruits and vegetables act as potential sources for the spread of various infectious diseases [2, 3, 6, 7].

Vegetables can be contaminated while growing in fields due to use of organic fertilizer or contaminated irrigation water or during harvesting, processing, distribution, sale and during consumption [8]. As a result, they have been involved in food-borne outbreaks [9]. Infective stages of intestinal parasites (IPs) are the most common contaminants of vegetables, as they are abundant in the environment [10]. Parasites such as *Cryptosporidium species (*spp), *Giardia lamblia (G.lamblia), Entamoeba histolytica (E.histolytica)*, *Ascaris lumbricoides (A.lumbricoides),* hookworms, *Enterobius vermicularis (E.vermicularis)*, *Trichuris trichiura (T.trichiura), Toxocara* spp., *Hymenolepis* spp., *Taenia* spp., *Fasciola* spp. and members of the family Trichostrongylidae could infect humans [11]. Hence, globally, an estimated 3.5 billion people are infected, and 450 million are sick from intestinal parasite infections (IPIs), with an estimated 200,000 deaths each year [12]. Intestinal parasites cause significant morbidity and mortality throughout the world, especially in tropical and sub-tropical countries [13]. Infection with IPs has been known to cause iron deficiency anemia, growth retardation in children and other physical and mental health problems [14, 15].

An effective way of selecting appropriate intervention steps to reduce pathogenic micro-organisms on vegetables is to identify sources of contamination and ecology of the pathogens [16]. However, in developing countries including Ethiopia, the medical impact of food-borne diseases is not known or under estimated because of absence of routine diagnosis and monitoring for the etiologic agents of food-borne infections [17]. The risk has been reported to be higher in towns, where there is poor hygienic and sanitary practice accompanied with overcrowding [5, 13].

Vegetables may get exposed to parasite contaminants in the pre-harvest (cultivation, irrigation, livestock manure) and post-harvest handling (storage, transportation) phases or while processing for consumption [18, 19]. Use of insufficiently treated wastewater and improperly composted manures are responsible for the high rates of parasitic contamination [18, 20]. Bad hygienic practice during production, transport, processing and preparation by handlers including consumers also contribute in vegetable contaminations [21].

Gamo Gofa Zone is known for the production of diverse types of fruits and vegetables that some cover the majority of country level consumption and others are distributed by retailers for local consumption. In Arba Minch (capital of the zone), it is common to see fruits and vegetables being sold in shops, at open markets and more commonly at road sides. It is also common to observe people buying those fruits and vegetables and eating in raw without washing. Despite this, the local health care units have no system to monitor the risks posed by unhygienic marketing and consumption of fruits and vegetables. A study conducted in Arba Minch town in 2014 showed a parasitic contamination rate of 54.4% [22]. Since then, efforts have been made to improve the water supply; sanitation and hygiene (WASH) of the population. Moreover national bi-annual deworming of risk population groups is being implemented since November 2015. Even though we suggest all these efforts indirectly decrease the contamination rate of vegetables, it should be supported by research based evidence. Therefore, this study was designed to determine the level of parasitic contamination of selected vegetables and associated factors in local markets of Arba Minch Town, Ethiopia.

## Methods

### Study design and area

A cross-sectional study was conducted from January to March 2018 among markets located in Arba Minch Town. Arba Minch is the capital of Gamo Gofa zone found in the Southern Nations Nationalities and Peoples Region, Southern Ethiopia. It is located at low altitude (1200–1300 m above sea level). The hot climate (annual temperature of 29.7 °C) makes the area to be conducive for cultivation of fruits and vegetables like Banana, Mango, cabbage, salad, carrot and many others [23].

### Sample collection

A pre-tested structured questionnaire was used to collect data about pre-disposing factors for parasitic contamination of vegetables at local markets. The questionnaire was prepared in English and translated to Amharic. The Amharic version was administered to vegetable vendors by face to face interview. About 0.2kgs of vegetables were purchased at four local markets (Sikela, shecha, Yetnebersh and Konso sefer). A total of 100 *Lycopersicon esculentum* (tomato), 96 *Brassica oleracea* (cabbage), 66 *Capsicum annuum* (Green pepper), 62 *Daucus carota* (carrot) and 23 *Lactuca sativa* (salad)*,* totally 347, samples were purchased and transported through transporting cold box to the Medical Parasitology Teaching Laboratory of Bahir Dar University. All the samples were processed and examined for parasitological contamination within 24 h of collection.

### Sample processing

A 100 g of each vegetable was chopped and immersed in 500 ml of physiological saline in graduated beaker. The contents were thoroughly agitated using a shaker in order to facilitate adequate washing.

The washing solution was then allowed to stand on the bench for overnight to allow time for proper sedimentation. Then a sediment for microscopic examination was prepared following a procedure explained elsewhere [24]. About 100 μl sediment was transferred to a clean glass slide, covered with cover slide and examined under light microscope using × 10 and × 40 objectives. Two slide preparations were examined for each sediment just to increase the sensitivity of the test. Another preparation of the sediment was examined by adding a drop of Lugol’s iodine before adding cover slide in order to easily detect parasite ova/cysts. Modified Zeihl-Neelsen staining technique was used for identification of oocysts of *Cryptosporidium*, *Isospora* and *Cyclospora* species as described elsewhere [25].

### Statistical analysis

Data collected from questionnaire and results of laboratory investigations were cleaned, entered and analysed using SPSS version 20.0. The percentage of vegetables contaminated with different types of medically important parasites were calculated. Chi-square test and logistic regression analysis were used to identify factors associated with parasitic contamination. Association between variables was considered statistically significant only if *P*-value ≤ 0.05 at 95% confidence level. The strength of association was measured through adjusted odds ratios (AOR).

## Results

A total of 347 vegetable samples were collected from local markets and examined for parasitological contamination. Vegetables were collected from four local markets namely Sikela (66.3%), Shecha (16.4%), Konso sefer (10.4%) and Yetnebersh (6.9%). Results of parasitological investigation showed that 87 vegetable samples were microscopically positive for at least one parasite. This gives an overall contamination rate of 25.1% (95%CI, 21.0–29.7). Among a total of 87 contaminated samples, 61 (70.1%) and 26 (29.9%) were contaminated with one and two parasite species respectively (Table 1).

**Table 1:** Frequency distribution of parasitic contamination among vegetables sold in local markets of Arba Minch Town from January to March 2018

Vegetable Type	Number Examined	Number positive (%)	Number of parasitic species detected (%)	
			One	Two
Tomato	100	35 (35.0)	22(22)	13(13)
Cabbage	96	23(24.0)	19(19.8)	4(4.2)
Green pepper	66	7(10.6)	6(9.1)	1(1.5)
Carrot	62	18(29.0)	12(19.4)	6(9.6)
Salad	23	4(17.4)	2(8.7)	2(8.7)
Total (%)	347	87(25.1)	61(17.6)	26(7.5)

The species and stages of parasites detected were ova of *A. lumbricoides* and hookworms*;* cysts of *G. lamblia, E. histolytica/dispar* and *Balantidium coli* (*B. coli)*; oocysts of *Cryptosporidium* and *Cyclospora* species; and larvae of *Strongyloides* like parasite. Cyst of *E. histolytica/dispar* (8.4%) was the most frequently detected parasite followed by cyst of *G. lamblia* (6.9%) and oocyst of *Cryptosporidium* species (5.8%). Among the five vegetable types included in the study, *E. histolytica/dispar* was detected only in two vegetables namely tomato (22/29) and carrot (7/29). On the contrary, *G. lamblia* was abundantly detected in samples of cabbage (16/24) followed by green pepper and carrot (4/24 in each) (Table 2).

**Table 2:** Prevalence of parasites among vegetables sold in local markets of Arba Minch Town from January to March 2018

Vegetable Type	Number Examined	Number Contaminated with								Total contaminated (%)
		*E.histolytica*	*G.lamblia*	*Cryptosporidium spp*	*B. coli like spp*	*A.lumbricoides*	*Cyclospora spp*	Hookworms	*Strongyloides* like	
Tomato	100	22	0	9	0	6	4	3	4	35 (35.0)
Cabbage	96	0	16	0	4	0	0	4	3	23(24.0)
G.pepper^*^	66	0	4	2	6	0	2	0	0	7(10.6)
Carrot	62	7	4	7	4	3	0	0	0	18(29.0)
Salad	23	0	0	2	1	0	1	0	0	4(17.4)
Total (%)	347	29(8.4)	24(6.9)	20(5.8)	15(4.3)	9(2.6)	7(2)	7(2)	7(2)	87(25.1)

^*^ *G.pepper = green pepper*

### Factors associated with parasitic contamination of vegetables

As analyzed using the chi-square (X^2^) test, kind of vegetables were significantly associated with parasitic contamination (*p* = 0.009). Tomato (35%) was the most frequently contaminated vegetable followed by carrot (29%), cabbage (24%), salad (17.4%) and green pepper (10.6%) (Table 3). Binary logistic regression analysis also showed that, as compared to green pepper, tomato was significantly less contaminated (AOR: 0.436; 95% confidence interval (CI): 0.192–0.991). Among 347 interviewed vendors, 290 have attended primary school while 36 and 21 have no formal education and secondary/above respectively. However, educational status of vendors was not significantly associated with parasitic contamination of vegetables (*p* = 0.086). The majority of vendors (74.9%) responded that they purchase vegetables directly from farmers while the rest (25.1%) are supplied by large scale vendors. These sources of vegetables were significantly associated with parasitic contamination level (*p* < 0.001) indicating that vegetables directly supplied by farmers were 3.547 times at higher risk of contamination as compared to those distributed by large scale vendors (AOR: 3.547; 95%CI: 2.066, 6.109) (Table 4).

**Table 3:** chi-square test of factors associated with parasitic contamination of vegetables sold in local markets of Arba Minch Town from January to March 2018

Variables	Category	Number examined	Rate of parasite contam inated N (%)	X^2^	P-value
Vegetable type	*Tomato*	100	35 (35.0)		
	*Salad*	23	4(17.4)		
	*Cabbage*	96	23(24.0)	13.484	0.009
	*Carrot*	62	18(29.0)		
	*Green pepper*	66	7(10.6)		
Washed before display	Yes	49	19(38.8)	5.703	0.117
	No	298	68(22.8)		
Market	Sikela	230	48(20.9)		
	Yetnebersh	24	12(50.0)	1.465	0.210
	Shecha	57	15(26.3)		
	Konso sefer	36	12(33.3)		
Means of display	On the floor	338	84(24.9)	0.336	0.562
	On table/shelf	9	6		
Handled by vendor who has	No formal edu	36	6(16.7)		
	Primary school	290	72(24.8)	4.899	0.086
	Secondary/above	21	9(42.8)		
Source of vegetables	Farmers	260	48(18.5)	24.122	0.000
	Large scale vendors	87	39(44.8)

**Table 4:** Binary logistic regression of factors associated with parasitic contamination of vegetables sold in local markets of Arba Minch Town from January to March 2018

Variables	Category	Number examined	Rate of parasite contam N (%)	Crude Odds Ratio (95%CI)		*P*-value	AOR (95%CI)	*P*-value
Vegetable type	*Tomato*	100	35 (35.0)	0.22(0.091,0.534)		0.01	0.321(0.109,0.939)	0.038
	*Salad*	23	4(17.4)	0.564(0.149,2.137)		0.399	1.200(0.320,4.504)	0.787
	*Cabbage*	96	23(24.0)	0.377(0.151,0.938)		0.036	0.533(0.177,1.608)	0.264
	*Carrot*	62	18(29.0)	0.290(0.111,0.755)		0.011	0.352(0.113,1.101)	0.073
	*Green pepper*	66	7(10.6)	1				
Washed before display	Yes	49	19(38.8)	1				
	No	298	68(22.8)	2.142(1.135,4.043)		0.019	0.534(0.191,1.493)	0.232
Market	Sikela	230	48(20.9)	1.896(0.884,4.064)		0.100	2.117(0.783,5.727)	0.139
	Yetnebersh	24	12(50.0)	0.500(0.173,1.441)		0.199	0.972(0.278,3.398)	0.965
	Shecha	57	15(26.3)	1.400(0.564,3.477)		0.469	0.953(0.302,3.001)	0.934
	Konso sefer	36	12(33.3)	1				
Means of display	On the floor	338	84(24.9)	1.512(0.370,6.178)		0.565		
	On table/shelf	9	6	1				
Handled by vendor who has	No formal edu	36	6(16.7)	3.750(1.095,12.842)		0.035	2.864(0.645,12.716)	0.166
	Primary school	290	72(24.8)	2.271(0.919,5.610)		0.076	1.088(0.318,3.726)	0.893
	Secondary/above	21	9(42.8)	1				
Source of vegetables	Farmers	260	48(18.5)	3.589(2.121,6.072)		0.000	3.547(2.066,6.109)	0.000
	Large scale vendors	87	39(44.8)	1				

## Discussion

In recent years, the federal ministry of health, in collaboration with concerned non-governmental organizations, is showing strong commitment to combat the public health impact of human parasites. However, the burden of intestinal parasitosis is still unacceptably high due to favorable climatic conditions, unsanitary conditions and little awareness by the community about the transmission and prevention of parasitic diseases. Open defecation is common in Ethiopia which, in turn, contaminates raw edible fruits and vegetables as well as water [26].

The overall parasitic contamination rate in the present study was 25.1% which is in line with findings from Nigeria and Iran [10, 27]. However it is lower than many of the previous findings elsewhere [22, 28–34]. On the other hand, it is higher as compared to studies from Iran, Turkey, Maiduguri (Nigeria) and Sudan [34–36]. These variations between the present study and previous results might be due to variations in geographical locations, climatic and environmental conditions, the kind of fruit and vegetable samples examined and number of fruits/vegetables examined, laboratory methods used, and sanitary status of the community.

As compared to similar study conducted in Arba Minch town in 2014, the present study showed a decrease in contamination rate by more than half (54.4% vs 25.1%) [22]. These differences might be due to ongoing bi-annual deworming program targeting helminthiasis. Moreover, improvements in the implementation of WASH program accompanied with continuous health education by the health extension workers to avoid open defecation might also play an important role. Differences in kind and number of fruits and vegetables assessed could also be responsible for the discrepancy.

In this study, tomato (35.0%) was the most frequently contaminated vegetable. On the contrary, tomato was the least contaminated vegetable according to previous studies [30, 32]. Leafy vegetables and/or those with uneven surfaces (salad, cabbage) make the parasitic stages more easily attach to their surfaces unlike to those having smooth surface (green pepper, tomato) [37]. Our finding is against the logic because we didn’t include equal number of the vegetables from each kind and very few numbers of samples were examined especially for salad. However, compared to green pepper, tomato was 32.1% less likely to be contaminated with parasites, similarly with study findings in Nigeria [10].

In the present study, cyst of *E. histolytica/dispar* (8.4%) was the most frequently detected parasite followed by *G. lamblia* (6.9%) which is in line with previous finding from Sudan [33]. However it varies among many other studies [10, 30–33, 35]. Iodine wet mount was examined in the present study which increases the sensitivity of stool microscopy for detection of protozoa cysts. Ova of *A.lumbricoides* were the predominant contaminants according to previous similar study done in Arba Minch. The national deworming program against soil transmitted helminths might bring this shift. In addition to this, variation in the season and kind of fruits and vegetables considered also matters.

*Cryptosporidium spp* (5.8%) was the third most common parasitic contaminant in the present study. However, the rate of contamination was lower as compared to findings from Ghana (17%), Egypt (29.3%) and Jimma, Ethiopia (12.8%) [4, 7, 30]. It is common to use animal excreta as fertilizer in the study area that we had expected higher rate of *Cryptosporidium* contamination. However, variations in the laboratory methods used might bring this variation as we used only ziehl Neelson staining technique.

Double contamination by two parasite species was observed in all kinds of vegetables (7.5%) but the rate of double infection is much lower as compared to studies conducted in different parts of Ethiopia. In addition, unlike to the previous studies, no more than two parasites have contaminated the same vegetable sample at a time [22, 26]. This is an indication that the level of contamination of vegetables is decreasing owing to implementation of parasite control activities in the country.

Vegetables directly supplied by farmers to vendors were 3.5 times more likely to be contaminated with parasites as compared to vegetables supplied by large scale vendors. Large scale vendors receive vegetables on the farm land, pack and transport to Arba Minch town using their vehicle, store it properly and distribute to small scale vendors. On the other hand, small scale vendors who receive vegetables directly from farmers transport it either via back of animals or human labor; vegetables are readily exposed to contamination in this case.

In the present study, display of vegetables without washing was not significantly associated with parasitic contamination (*p* = 0.232) with probable reason of few number of washed vegetables included in the study (*n* = 49). However, previous studies in Turkey and Iran showed that washing of vegetables contributes for significant decrease in the rate of parasitic contamination [37, 38]. According to study results by Fallah et al, traditional washing with tap water reduces rate of parasitic contamination (32.6% vs 1.3%) but does not totally remove parasites from vegetables. Pre-washing could not inactivate viable infectious parasitic stages. Hence, in order to totally remove parasites, vegetables need to be washed following standard technique using chemicals like calcium hypochlorite and automated fruit-vegetable washer [38].

Previous studies have identified that means of display, type of water used for washing before display and contamination extent of irrigation water were significantly associated with parasitic contamination of vegetables [10, 30, 34]. In order to identify potential sources or factors which contribute for parasitic contamination of vegetables, large scale follow up study starting from farm to in-house consumption is recommended.

## Conclusion

The present study reveals that parasitic contamination rate in vegetables sold at Arba Minch local markets is significantly considerable. *E. histolytica/dispar* was the most frequently detected parasite. Vegetable types were determinant factors for variations in rate of contamination that tomato was the most commonly contaminated vegetable. Vegetables directly supplied by farmers to vendors were more prone for contamination as compared to those supplied by large scale vendors. We recommend to the local public health sector to establish a system for continuous monitoring of contamination of vegetables sold at local markets. The public health sector should also advocate to the community not to consume vegetables without adequate washing or proper cooking. Finally we call up further study considering seasonal variations in order to exhaustively assess associated factors.

## Abbreviations

AOR: Adjusted odds ratio
CI: Confidence interval
IPIs: Intestinal parasitic infections
IPs: Intestinal parasites
WASH: Water sanitation and hygiene
